# Anti-BCMA and GPRC5D bispecific antibodies in relapsed/refractory primary plasma cell leukemia: a case report

**DOI:** 10.3389/fimmu.2024.1495233

**Published:** 2024-11-29

**Authors:** Chiara Bernardi, Yan Beauverd, Thien An Tran, Marie Maulini, Maria Mappoura, Sarah Morin, Federico Simonetta, Anne Cairoli, Holger W. Auner, Kaveh Samii, Yves Chalandon, Carmen de Ramon Ortiz

**Affiliations:** ^1^ Division of Haematology, Department of Oncology, Geneva University Hospitals, Geneva, Switzerland; ^2^ Translational Research Center for Oncohematology, Department of Medicine and Department of Pathology and Immunology, Faculty of Medicine, University of Geneva, Geneva, Switzerland; ^3^ Division of Haematology, Department of Oncology, University Hospital of Lausanne (CHUV), Lausanne, Switzerland

**Keywords:** bispecific Ab, plasma cell leukemia (PCL), BCMA, GPRC5D, elranatamab, talquetamab

## Abstract

Plasma cell leukemia (PCL) is an aggressive and high-risk variant of multiple myeloma (MM) with a very poor prognosis. Given its rarity and aggressiveness, there is a lack of clinical trials testing the efficacity of novel therapies in these patients. New immune approaches such as B-cell maturation antigen (BCMA) and G protein-coupled receptor, family C, group 5, member D (GPRC5D) -targeting agents, including chimeric antigen receptor (CAR) T-cells and bispecific antibodies could play a role in PCL treatment. However, PCL patients were excluded from recent pivotal clinical trials testing those agents and only some case reports have been published. We present here the clinical course of a patient with relapsed/refractory (R/R) primary (p) PCL who was treated with anti-BCMA and anti-GPRC5D bispecific antibodies at our center.

## Introduction

Plasma cell leukemia (PCL) is a rare and aggressive disease defined by the presence of 5% or more circulating plasma cells in peripheral blood smears in patients otherwise diagnosed with multiple myeloma (MM) (1). PCL is further classified as primary (pPCL; 60% of cases) when occurring *de novo* or secondary (sPCL; 40% of cases) when it represents a transformation from a previously known plasma cell disorder (1). Patients with pPCL are younger than MM patients and often have a higher tumor burden, extramedullary involvement, high levels of LDH, cytopenias and poor-risk cytogenetic abnormalities (2). Given the rarity and aggressiveness of the disease, there is a lack of clinical trials testing the efficacity of novel therapies in these patients. Only some trials, such as the GMMG-CONCEPT, include patients with pPCL (3). Currently, there is no established standard of care. The survival of patients with pPCL has only mildly improved in the last decade with the use of novel agents, such as immunomodulators, proteasome inhibitors, monoclonal antibodies targeting CD38 in conjunction with stem cell transplantation but its prognosis remains very poor (1, 4, 5). New immune approaches such as chimeric antigen receptor (CAR) T-cells and bispecific antibodies could play a role in PCL treatment. However, PCL patients were excluded from recent pivotal clinical trials testing those agents and only some case reports have been published (6, 7). A phase 1 study shows a potential benefit of CAR-T cell in patients with pPCL with a short duration of response (6). However, given the time needed for CAR-T cell manufacturing, the disease often progresses too quickly to allow for CAR-T cells delivery. Bispecific antibodies have the advantage of being *off-the-shelf*, potentially representing a more interesting treatment option for these patients. Here we present the clinical course of a patient with pPCL who was treated with BCMA and GPRC5D bispecific antibodies.

## Case description

**Figure 1:**
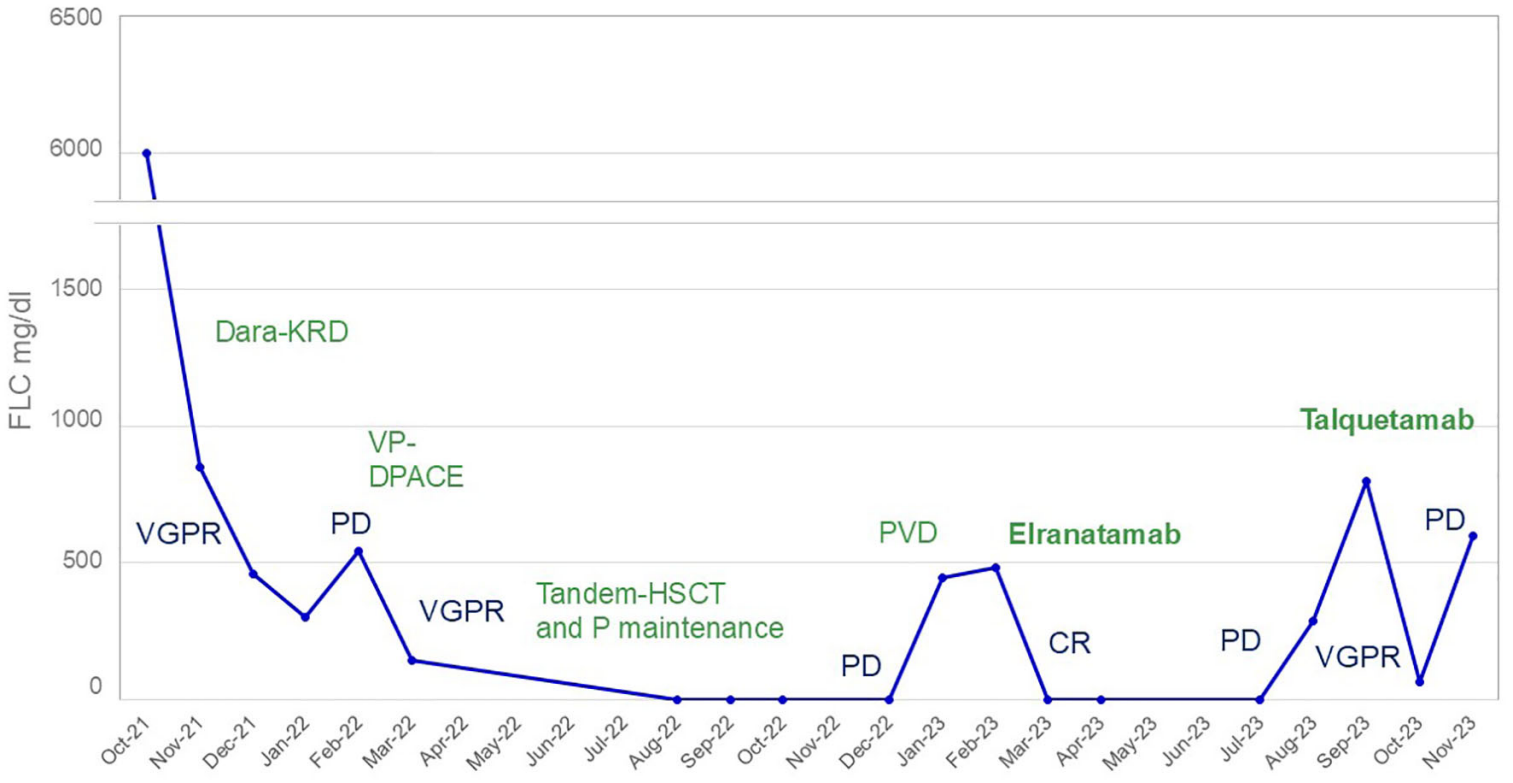
Free lambda light chain evolution during treatment. In blue: response to treatment; in green: treatment. Dara-KRD, daratumumab, carfilzomib, lenalidomide and dexamethasone; HSCT, hematopoietic stem cell transplantation; P, pomalidomide; PVD, pomalidomide, bortezomib, dexamethasone; VP-DPACE, dexamethasone, pomalidomide, cisplatin, doxorubicin, cyclophosphamide, bortezomib and etoposide; CR, complete response; PD, progressive disease; VGPR, very good partial response.

**Figure 2:**
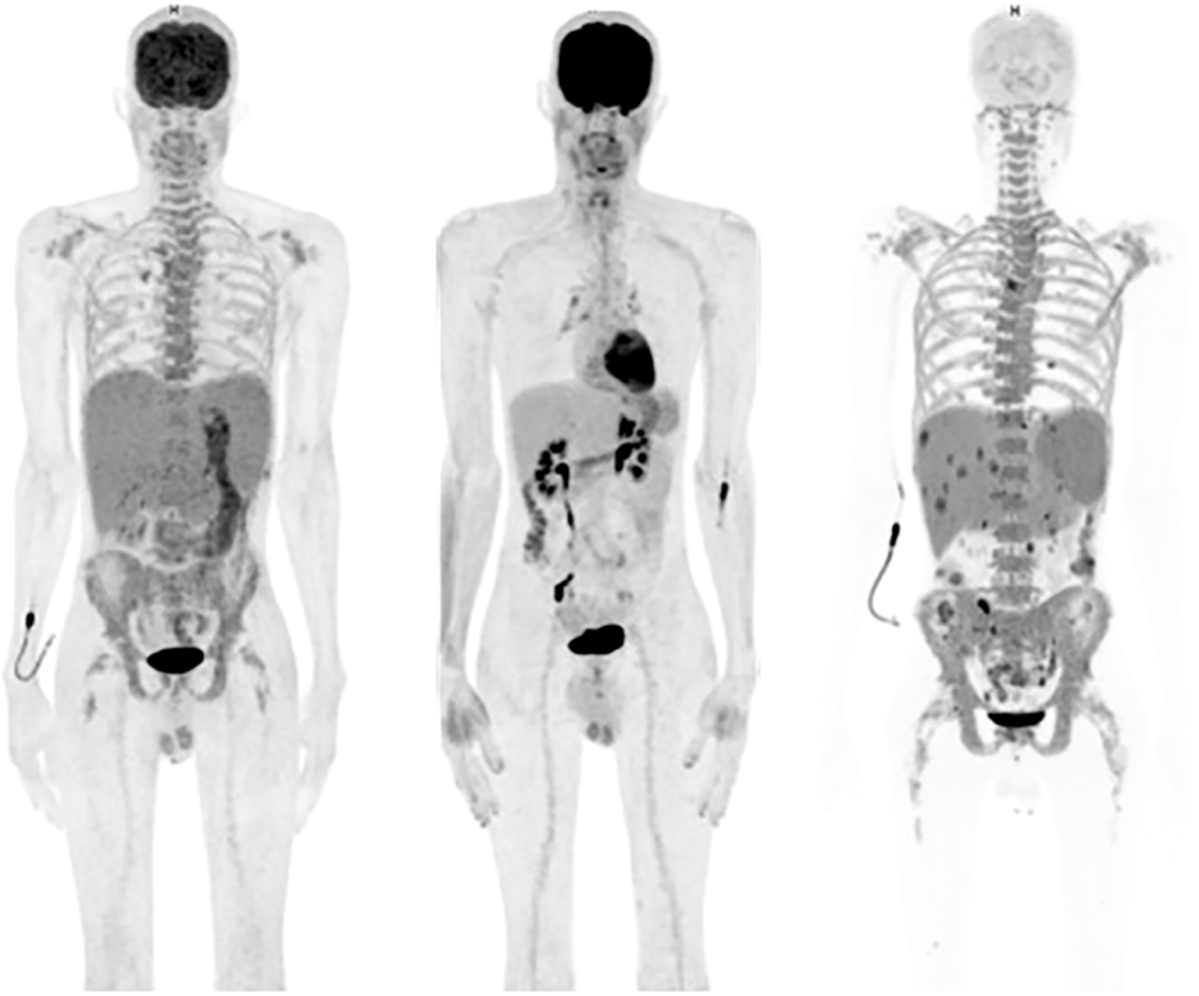
Left: initial positron emission tomography/computed tomography (PET/CT) imaging showing hepatosplenomegaly, a diffuse hypermetabolism of the bone marrow and the stomach; middle: PET/CT at day 100 after tandem HSCT showing no pathological hypermetabolism; right (patient in hypoglycemia): PET/CT at the moment of the last progression after Talquetamab showing an explosive disease with hypermetabolic hepatosplenomegaly and multiple hypermetabolic lesions in the liver and spleen and hypermetabolism of the axial skeleton.

A 40-year-old male presented with bone pain. Initial laboratory investigations (Oct-21) showed severe anemia with hemoglobin at 7.6 g/dL and thrombopenia at 16 G/l. White blood cells count was at 15.1 G/l with 47% circulating plasma cells, hypercalcemia, elevated LDH and β2-microglobulin levels. Serum protein electrophoresis showed a γ- and - β migrating paraprotein quantified at 26.6 g/l with a monoclonal IgA lambda at serum protein immunofixation (IgA level of 31.6 g/l and free lambda chains (FLC) at 6730 mg/l). Bone marrow (BM) aspirate showed an infiltration by plasma cells constituting 80%. Chromosomal karyotyping revealed high-risk cytogenetics with complex karyotype and loss of 17p13.1 region. FISH analysis revealed t(14;16). PET-CT imaging showed increased gastric and BM activity. Histology of a gastric biopsy confirmed gastric infiltration by monoclonal plasma cells. Lumbar punction was negative. The patient was diagnosed with pPCL. He received induction therapy consisting of 4 cycles of KRD therapy (carfilzomib; lenalidomide and dexamethasone) (8), with addition of weekly subcutaneous (SC) 1800mg Daratumumab to the cycle 3 and 4, after approval of insurance reimbursement (5). After cycle 1, the patient achieved a very good partial response (VGPR) (9), but he quickly progressed after 4 cycles (**Figure 1**). Second line therapy with VP-DPACE was started (Feb-22) (dexamethasone, cisplatin, doxorubicin, cyclophosphamide, etoposide; bortezomib and pomalidomide) (10), resulting in VGPR after 2 cycles, followed by tandem autologous hematopoietic stem cell transplant (HSCT) after a melphalan based-regimen (200mg/m^2^) and maintenance therapy with Pomalidomide from day +30. At day 100 post-second autologous HSCT (Dec-22), he experimented a biochemical progression. He received one cycle of PVD (OPTIMISMM protocol: bortezomib, dexamethasone and pomalidomide) (11), but was refractory with rapidly progressive disease. Fourth line treatment with Elranatamab, an anti-BCMA bispecific antibody, was started (Feb-23) (SC Elranatamab 76mg once weekly in a 28-d cycle). After two step-up priming doses of 12mg and 32mg given on day 1 and day 4 (12), he presented a grade 2 CRS (cytokine release syndrome) and grade 4 neutropenia, requiring treatment with IV Tocilizumab 8mg/kg. grade 1 CRS and hematologic toxicity grade 4 was observed after the second step-up dose. CR was achieved after 1 cycle of treatment of Elranatamab and a total of 5 cycles were administered. Patient had an ECOG of 0 and travelled regularly. Monthly intravenous immunoglobulin replacement and antibiotic prophylaxis with trimetoprim-sulphametoxazol and valaciclovir 500mg bid were administered throughout treatment. After cycle 5 (Jul-23), a biochemical progression was observed, with a significant rise in FLC and a BM aspirate showing a 20% infiltration with monoclonal plasma cells. A 5^th^ line was started with a GPRC5D bispecific antibody, Talquetamab (Sept-23) (13). During the first cycle (SC, ramp up doses of 0.01 mg/kg at day 1, 0.06 mg/kg at day 3, 0.4 mg/kg at day 5 and 7) patient presented grade 1 CRS. Because of persistent fever, a dose of IV Tocilizumab 8mg/kg was administered at day 8. After 2 cycles of Talquetamab (Oct-23), the patient presented an explosive relapse (**Figure 2**), with a rapid rise in FLC and clinical and radiologic relapse with multiple bone lesions on PET-CT. Palliative care was offered to the patient and he died 3 weeks later.

## Discussion

During the last few years, the outcome of MM patients has dramatically improved with the addition of novel agents. However, this is not the case for pPCL, and early relapses are still observed and prognosis remains dismal (2). Novel T cell-engaging immunotherapies, such as bispecific antibodies and CAR T-cell have not yet been tested in PCL patients. Our experience evidences the potential feasibility of bispecific antibodies use in this disease with no limiting toxicities and a clear advantage of *off-the-shelf* in this rapidly progressive and aggressive disease. Our case experienced a very quick achievement of response, after only 1 cycle, and allowed the patient an improved quality of life. However, duration of response was short, 5 months on Elranatamab and 2 months on Talquetamab. Potential strategies to increase duration of response could be to use bispecific antibodies at earlier lines of therapies or as a maintenance therapy after HSCT. Bispecific antibodies could also represent an interesting bridging therapy before anti-BCMA CAR T cells, especially Talquetamab given its different antigen target. Given the extremely poor prognosis of PCL and the lack of data in this setting, prospective clinical trials for PCL patients are needed.

## Data Availability

The original contributions presented in the study are included in the article/supplementary material. Further inquiries can be directed to the corresponding authors.
